# TRAPPC4-ERK2 Interaction Activates ERK1/2, Modulates Its Nuclear Localization and Regulates Proliferation and Apoptosis of Colorectal Cancer Cells

**DOI:** 10.1371/journal.pone.0023262

**Published:** 2011-08-03

**Authors:** Shu-Liang Zhao, Jie Hong, Zuo-Quan Xie, Jie-Ting Tang, Wen-Yu Su, Wan Du, Ying-Xuan Chen, Rong Lu, Dan-Feng Sun, Jing-Yuan Fang

**Affiliations:** 1 Department of Gastroenterology, Shanghai Jiao-Tong University School of Medicine Ren-Ji Hospital, Shanghai Institute of Digestive Disease, Shanghai, People's Republic of China; 2 Division of Anti-tumor Pharmacology, State Key Laboratory of Drug Research, Shanghai Institute of Materia Medica, Chinese Academy of Sciences, Shanghai, People's Republic of China; Roswell Park Cancer Institute, United States of America

## Abstract

The trafficking protein particle complex 4 (TRAPPC4) is implicated in vesicle-mediated transport, but its association with disease has rarely been reported. We explored its potential interaction with ERK2, part of the ERK1/2 complex in the Extracellular Signal-regulated Kinase/ Mitogen-activated Protein Kinase (ERK-MAPK) pathway, by a yeast two-hybrid screen and confirmed by co-immunoprecipitation (Co-IP) and glutathione S-transferase (GST) pull-down. Further investigation found that when TRAPPC4 was depleted, activated ERK1/2 specifically decreased in the nucleus, which was accompanied with cell growth suppression and apoptosis in colorectal cancer (CRC) cells. Overexpression of TRAPPC4 promoted cell viability and caused activated ERK1/2 to increase overall, but especially in the nucleus. TRAPPC4 was expressed more highly in the nucleus of CRC cells than in normal colonic epithelium or adenoma which corresponded with nuclear staining of pERK1/2. We demonstrate here that TRAPPC4 may regulate cell proliferation and apoptosis in CRC by interaction with ERK2 and subsequently phosphorylating ERK1/2 as well as modulating the subcellular location of pERK1/2 to activate the relevant signaling pathway.

## Introduction

TRAPPC4, the human ortholog of yeast Trs23p, also known as synbindin, is generally known as a neuronal cytoplasmic protein originally identified by yeast two-hybrid screening using the syndecan-2 (belonging to a family of cell-surface heparan sulfate proteoglycans that regulates cell behavior through signal transduction pathways [1], [2], [3]) cytoplasmic domain as bait [4]. It is considered a physiological ligand of syndecan-2 on dendritic spines that is involved in syndecan-2 induced spine formation by recruiting intracellular vesicles toward post-synaptic sites in rat hippocampal neurons. TRAPPC4 has been detected in CD34+ hematopoietic stem/progenitor cells (HSPCs) and thus is also known as HSPC172.

TRAPPC4 as a member of the trafficking protein particle (TRAPP) family of proteins is implicated in vesicle-mediated transport, a process carried out by virtually every cell and is required for the proper targeting and secretion of proteins. At present, there are 10 known yeast TRAPP subunits (Bet5p, 3p, Trs20p, 23p, 31p, 33p, 65p, 85p, 120p, 130p), and higher eukaryotes have orthologs for eight of these (TRAPPC1∼5,6a,6b,8,9) [5]. Together, they form two types of mutisubunit complexes: TRAPP I and TRAPP II. In yeast, these complexes function in a number of processes, including endoplasmic reticulum-to-Golgi transport (TRAPP I) and an ill-defined step at the trans Golgi network (TRAPP II) [6], [7], [8]. Studies in normal rat kidney (NRK) cells and HeLa cells also showed that the TRAPP complex plays a role during ER-Golgi transport [9], [10]. The PDZL domain in TRAPPC4 is one of the most unique features of the vertebrate complex when compared with yeast TRAPP I. Dysfunction of TRAPP subunits have been implicated in human diseases. Mutations in TRAPPC1 (MUM-2) were reported to result in expression of antigenic peptides in melanoma [11], and mutations in TRAPPC2 (sedlin) have been linked to Spondyloepiphyseal dysplasia tarda (SEDT) [12]. However, the role of TRAPPC4 in disease has rarely been studied.

Colorectal cancer (CRC) is a significant cause of morbidity and mortality throughout the world [13]. Colorectal carcinogenesis is a complex multi-step process involving progressive disruption of intestinal epithelial-cell proliferation, apoptosis, differentiation and survival mechanisms [14]. The Extracellular Signal-regulated Kinase/ Mitogen-activated Protein Kinase (ERK-MAPK) pathway is one of the most important signal transduction pathways for cellular physiology, and several key growth factors and proto-oncogenes promote growth and differentiation through this cascade [15]. Upon activation, the ERK1/2 complex migrates to the nucleus where it phosphorylates various transcription factors that regulate genes to increase cell proliferation and modulate cell apoptosis [16]. However, the detailed mechanisms of activation and nuclear translocation of ERK1/2 have not been fully clarified.

In the current study, a yeast two-hybrid screen was performed to identify ERK1 and ERK2 binding proteins. TRAPPC4 was found to bind with ERK2. We confirmed the interaction and further investigated the role of the TRAPPC4-ERK2 interaction in CRC.

## Results

### TRAPPC4 was identified as an ERK2-interacting factor by two-hybrid screening

Phosphorylation and nuclear entry of ERK1/2 is critical for activation of the ERK-MAPK pathway. To identify factors related to the process, a yeast two-hybrid screen was performed with ERK1 and ERK2 as baits (full-length) in conjunction with a human HeLa MATCHMAKER cDNA library. Of the colonies growing on Leu-Trp-His plates that were screened, 57/61 were positive for ERK2 and 12/32 for ERK1 in a ß-galactosidase assay. Plasmids from these positive colonies were extracted, amplified in bacteria and co-transformed back into yeast along with the bait plasmids for further testing by ß-galactosidase assay. Eleven clones were confirmed to have a positive interaction with ERK2 and twelve with ERK1. Among these plasmids that were positive hits for ERK2 and ERK1, TRAPPC4 was identified in five and six of the sequences, respectively, after sequencing. This was the first time that TRAPPC4 was identified as a potential interacting protein of ERK2.

### Validation of the TRAPPC4-ERK2 interaction

**Figure 1:**
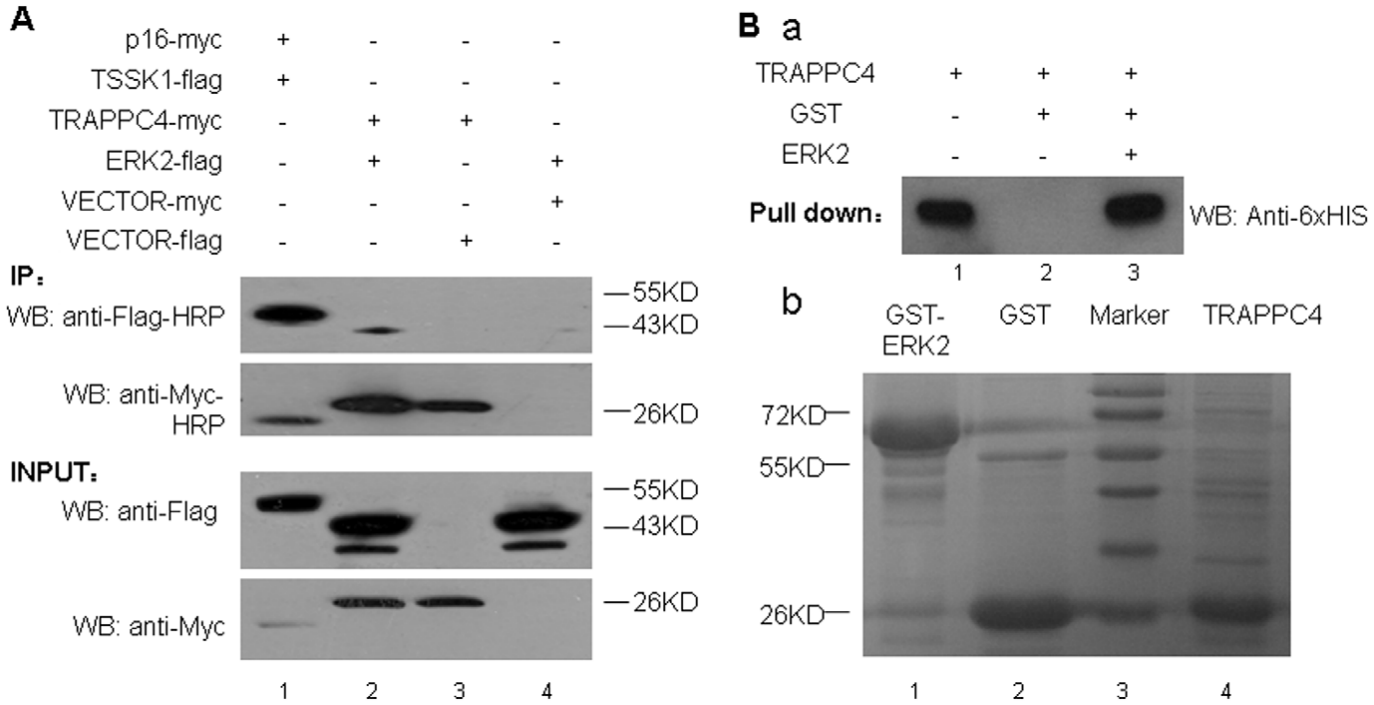
Identification of the protein-protein interaction between TRAPPC4 and ERK2 by co-immunoprecipitation and pull-down assay. A: Co-immunoprecipitation of TRAPPC4 and ERK2. 293T cells were transfected with empty pcDNA vector or pcDNA vector to express Flag tagged ERK2 (42 kD), or Myc tagged TRAPPC4 (26 kD). Cell lysates were subjected to immunoprecipitation with anti-Flag-HRP antibody or anti-Myc-HRP antibody. The presence of ERK2-Flag and TRAPPC4-Myc in cell extracts prior to immunoprecipitation was controlled using anti-Flag and anti-Myc antibodies (Input). Co-transfection of expression vectors for p16-Myc and TSSK1-Flag was used for the positive control (lane 1). B: Representative results of GST pulldown validation experiments. (a) Immunoblot analysis of GST, and GST-ERK2 proteins used as negative control (lane 2) and bait (lane3) for the pulldown assay. Proteins were detected using anti-6×HIS antibody following SDS-PAGE and membrane transfer. Lane 1 showed the blank control without GST or GST-ERK2. (b) ERK2 expressed as a GST fusion protein from bacteria was bound to beads and incubated with TRAPPC4 expressed as 6×His-TRAPPC4 fusion protein for a pulldown experiment. GST-ERK2 protein(lane1), GST protein(lane2) and TRAPPC4 protein(lane4) were detected by Coomassie staining. Lane3 showed the marker.

To further confirm the interaction between TRAPPC4 and ERK2, co-immunoprecipitation and GST pull-down experiments were conducted. For co-immunoprecipitation, full-length TRAPPC4 cDNA was obtained and cloned into pCDEF-Myc, while the ERK2 sequence was cloned into pCDEF-Flag. The resulting pCDEF-Myc-TRAPPC4 and pCDEF-Flag-ERK2 vectors were validated by sequencing and co-transfected into the 293T cell line. Western blot analysis (Fig. 1A) showed that the plasmids could express significant levels of the proteins. After using an anti-Myc antibody for co-immunoprecipitation, both the TRAPPC4-myc band and ERK2-flag band were detected from the pCDEF-Myc-TRAPPC4 and pCDEF-Flag-ERK2 co-transfected cells, indicating there is an interaction between TRAPPC4 and ERK2. In the positive control sample with co-transfected pCDEF-Myc-p16 and pCDEF-Flag-TSSK1 vectors, both the p16-Myc band and TSSK-flag band were detected, indicating that the proteins could be co-immunoprecipitated by our protocol based on the known interaction between p16 and TSSK1. Only the TRAPPC4-myc band was detected when pCDEF-Myc-TRAPPC4 was co-transfected with an empty flag vector, while no band was detected when pCDEF-Flag-ERK2 was co-transfected with empty Myc vector, suggesting that the interactions observed above are specific and not mediated by the protein tags.

As it is possible that the TRAPPC4 and ERK2 interaction may be indirect because other protein factors in the whole cell extract may be involved in mediating the interaction, e.g. acting as ‘bridging’ factors, we next examined a possible direct interaction between the two proteins using GST pull-down assays. TRAPPC4 was pulled down with GST-ERK2 fusion proteins (Fig. 1B, lane c), but not with GST alone (Fig. 1B, lane b), indicating that TRAPPC4 and ERK2 specifically and directly interacted *in vitro*.

### TRAPPC4 regulates ERK1/2 phosphorylation in the SW1116 cell line

**Figure 2:**
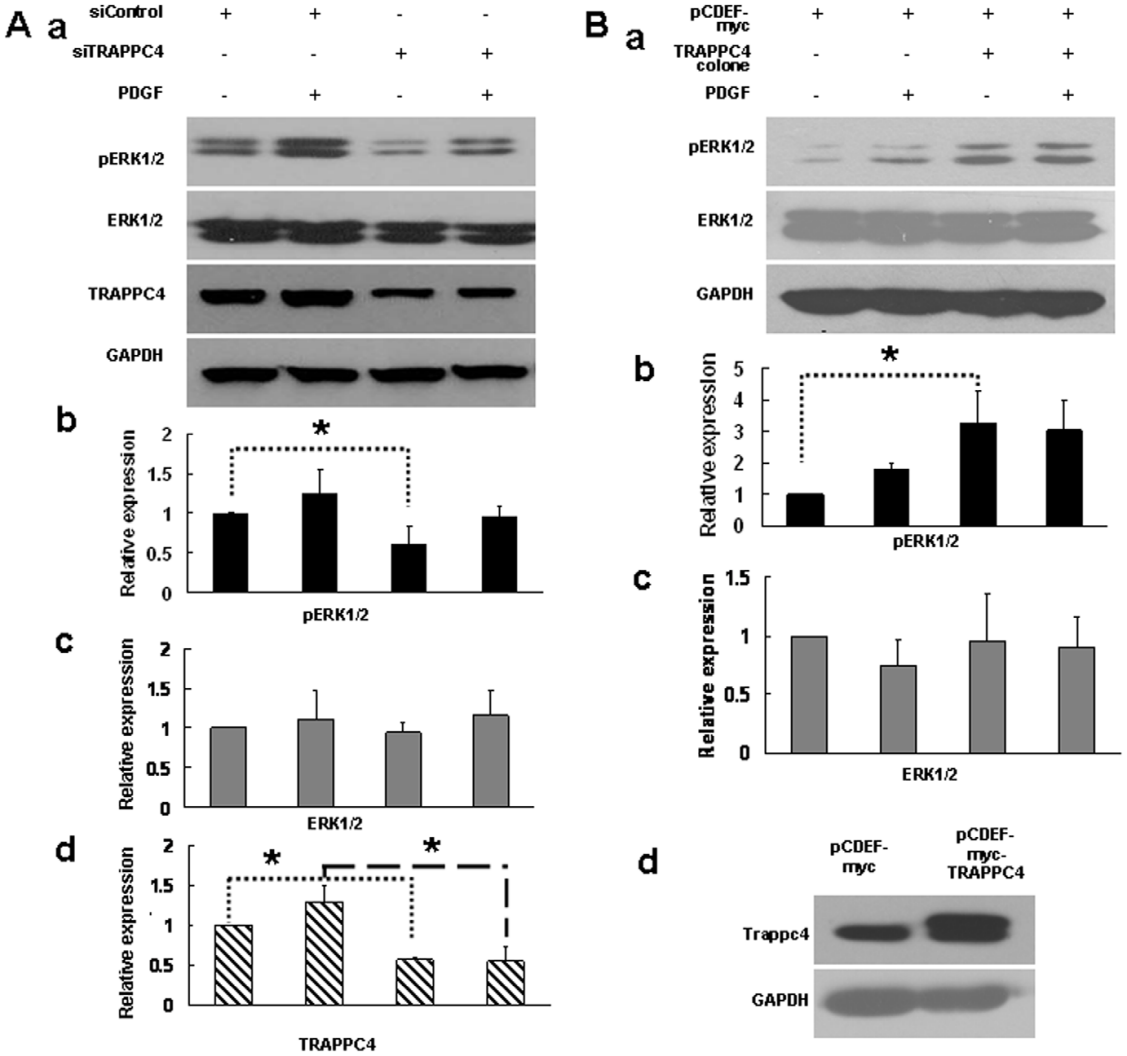
Expression of TRAPPC4 influences the activation status of ERK1/2 in CRC cells. Cells were lysed, and equal amounts of protein were analyzed by Western blot analysis using antibodies against phospho-ERK1/2 (pERK1/2), or ERK1/2, or TRAPPC4. The density of the Western blot bands were normalized to the amount of GAPDH protein. The data shown are representative of three replicate experiments. *,*p*<0.05. A. Cells were transfected with TRAPPC4 siRNA (siTRAPPC4) to knockdown TRAPPC4 expression or with Negtive Control siRNA (siControl) as control; Also cells were treated with PDGF to activate ERK-MAPK pathway, or with its solvent (PBS+0.1 BSA) as control. B. Cells were transfected with pCDEF-myc-TRAPPC4 vector to overexpress TRAPPC4 protein or with pCDEF-myc vector as control; Also cells were treated with PDGF to activate ERK-MAPK pathway, or with its solvent (PBS+0.1 BSA) as control.

Phosphorylation of ERK1/2 is a critical step in the ERK signaling pathway. Now that we had shown that TRAPPC4 interacted with ERK2, we next examined the relative contribution of TRAPPC4 in ERK1/2 phosphorylation. After reducing the expression level of TRAPPC4 in SW1116 cells by RNAi or overexpressing it by transfection of a plasmid encoding the full-length gene, we determined the change in pERK levels by Western blotting. Detection of GAPDH was used as a loading control. While there were no observed differences in the protein levels of total ERK1/2 in all the groups (Fig. 2), the phosphorylation level of ERK1/2 was down-regulated after siRNA -mediated depletion of TRAPPC4 (Fig. 2A) and up-regulated after its overexpression (Fig. 2B). Furthermore, we treated both groups of cells with platelet-derived growth factor (PDGF), one of the activators of the ERK-MAPK pathway, and found that the level of ERK1/2 phosphorylation after the treatment was not significantly different between the groups, despite their differences in TRAPPC4 levels (Fig. 2).

### TRAPPC4 regulates the spatial distribution of phosphorylated ERK1/2

Since we had shown that TRAPPC4 could influence the activation status of ERK1/2, we wanted to determine its effect on the subcellular localization of pERK1/2. Next, we compared pERK1/2 and ERK1/2 expression in the separated cytoplasmic and nuclear fractions when TRAPPC4 was depleted or overexpressed as described above. Again, GAPDH was used as a loading control for cytoplasmic protein input, and TBP was used as a control for nuclear protein input.

**Figure 3:**
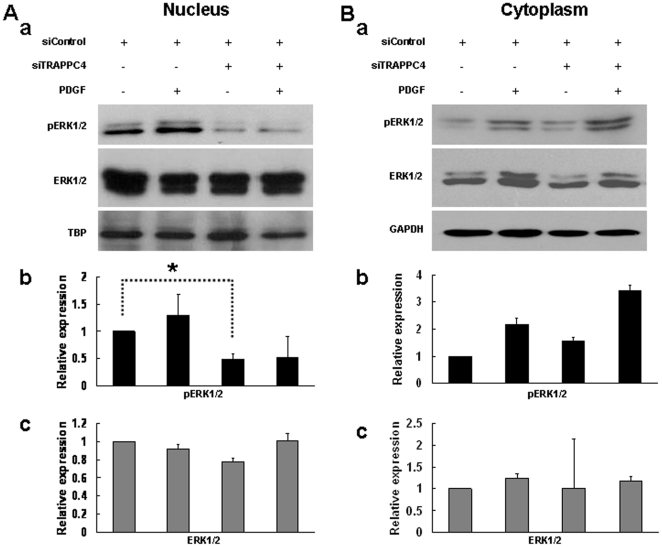
Knockdown of TRAPPC4 decreased actived ERK1/2 level in nucleus on SW1116 cell line. SW1116 cells were transfected with TRAPPC4 siRNA (siTRAPPC4) to knockdown TRAPPC4 expression or with Negtive Control siRNA (siControl) as control; Also cells were treated with PDGF to activate ERK-MAPK pathway, or with its solvent (PBS+0.1% BSA) as control. Cytoplasm and nuclear extracts were prepared as described in experimental procedures, and equal amounts of protein were analyzed by Western blot analysis using antibodies against phospho-ERK1/2 (pERK1/2), or ERK1/2, or TRAPPC4. The density of the Western blot bands were normalized to the amount of GAPDH protein for cytoplasma protein or TBP for nuclear protein. The data shown are representative of three replicate experiments. *,*p*<0.05.

**Figure 4:**
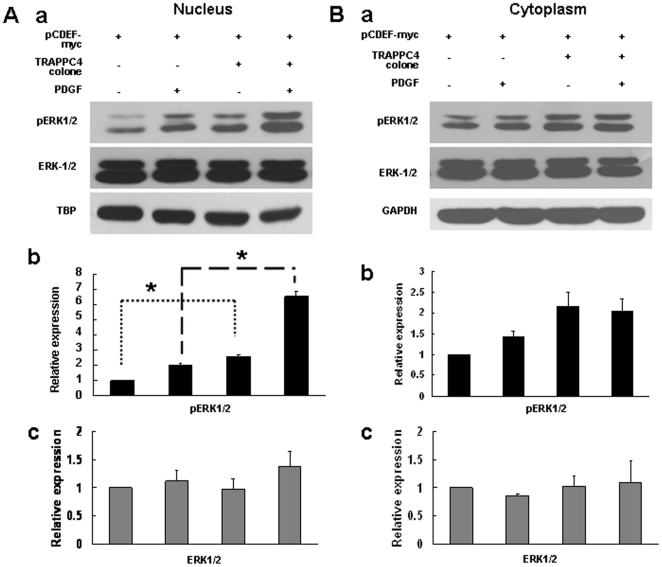
Overexpression of TRAPPC4 increased actived ERK1/2 level in nucleus on SW1116 cell line. SW1116 cells were transfected with pCDEF-myc-TRAPPC4 vector to overexpress TRAPPC4 protein or with pCDEF-myc vector as control; Also cells were treated with PDGF to activate ERK-MAPK pathway, or with its solvent (PBS+0.1% BSA) as control. Nuclear (A) and cytoplasm (B) extracts were prepared as described in experimental procedures, and equal amounts of protein were analyzed by Western blot analysis using antibodies against phospho-ERK1/2 (pERK1/2), or ERK1/2, or TRAPPC4. The density of the Western blot bands were normalized to the amount of TBP for nuclear protein or GAPDH protein for cytoplasma protein. The data shown are representative of three replicate experiments. *,*p*<0.05.

By Western blotting, we found that after TRAPPC4 siRNA treatment, the level of pERK1/2 significantly decreased in the nucleus (Fig. 3A). Meanwhile, no significant difference in pERK1/2 was found in the cytoplasm when quantified, even though a slight increase was suggested visually (Fig. 3B). On the other hand, when TRAPPC4 was overexpressed, pERK1/2 expression was upregulated significantly in the nucleus (Fig. 4A). While in the cytoplasm, our results showed a slight visual increase without significant differences when quantified (Fig. 4B).

PDGF can activate the ERK-MAPK pathway through receptor tyrosine kinases [17]. When the SW1116 cell line was stimulated with PDGF, a slight decrease of pERK1/2 was visually observed in the nuclear fraction after TRAPPC4 knock-down, but it was not significant upon quantification. Meanwhile, no significant alteration in pERK1/2 was quantified in the cytoplasmic fraction, even though a slight increase was suggested visually (Fig. 3). However, PDGF stimulation of cells overexpressing TRAPPC4 resulted in significant pERK1/2 upregulation in the nucleus, but not in the cytoplasm (Fig. 4).

### TRAPPC4 modulates proliferation and depletion of TRAPPC4 induces apoptosis in the SW1116 cell line

**Figure 5:**
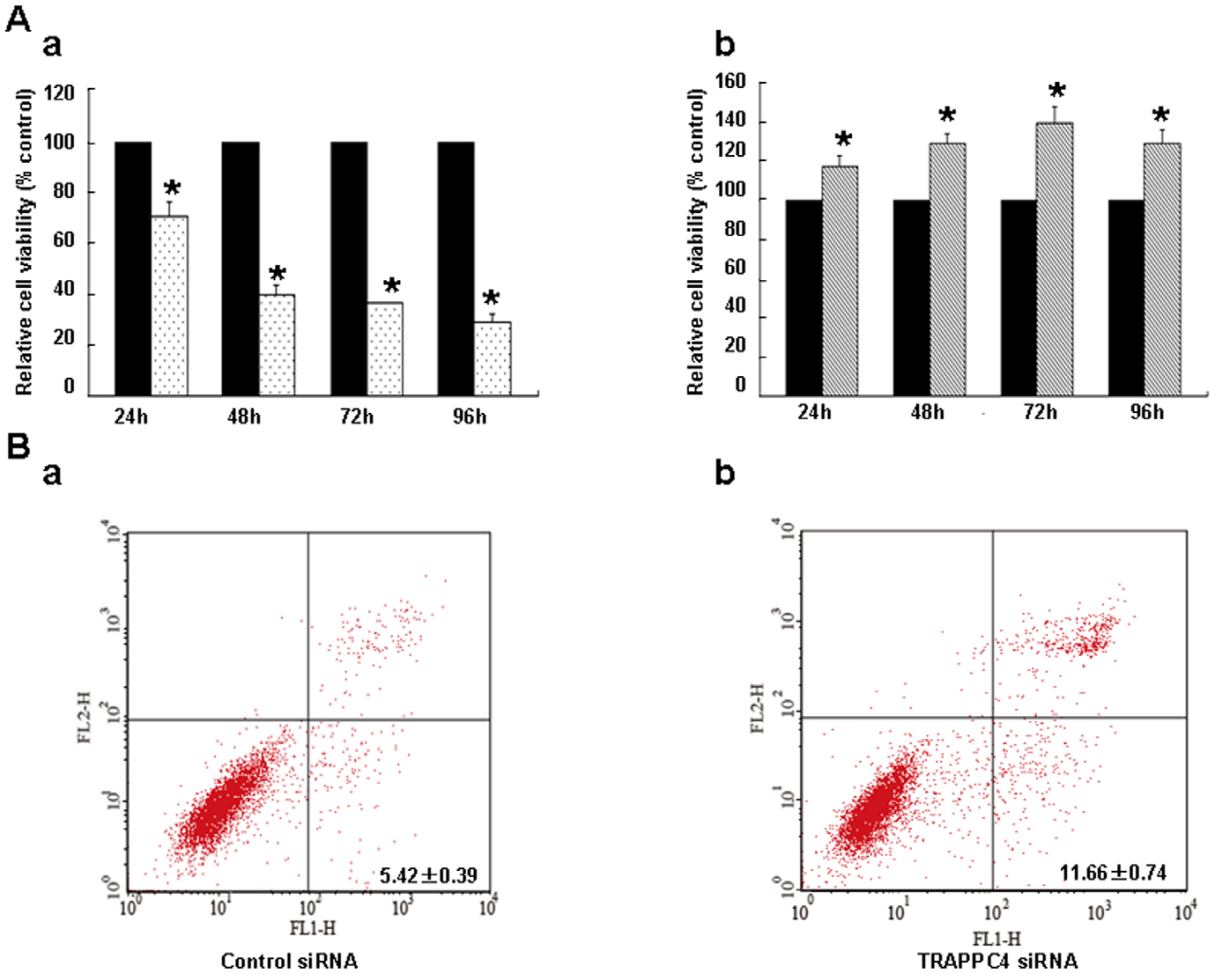
Expression of TRAPPC4 inflenced cell growth and its depletion induced apoptosis on SW1116. A: CCK-8 assay. SW1116 cells were seeded in a 96-well plate until subconfluent. Viable cells were determined by CCK-8 assay. Three independent time were performed in triplicate. Cells were treated with TRAPPC4 siRNA to knockdown TRAPPC4 expression or with Negtive Control siRNA used as control (a), as well as tranfered with pCDEF-myc-TRAPPC4 vector to overexpress TRAPPC4 protein or with pCDEF-myc vector as control (b). *, *p*<0.05. B: Apoptosis analysis. After SW1116 cells were treated with TRAPPC4 siRNA or Negtive Control siRNA used as control for 48 h. Apoptosis was carried out using the Annexin V assay with Propidium iodide counterstaining allowing quantification by flow cytometry. Three independent time were performed in triplicate. The data were presented as means ± standard deviation (SD). There was significant difference between the two groups( *p*<0.01).

The activation of ERK-MAP kinases has been linked to cell proliferation [16]. To further study the function of TRAPPC4 in CRC cells, we compared cell proliferation of SW1116 cells when TRAPPC4 expression was decreased by siRNA or over-expressed with full-length TRAPPC4 gene transfection with that of mock control. Results of the Cell Counting Kit (CCK)-8 assay revealed a significant inhibition of growth after TRAPPC4 depletion and an increase of cell viability when TRAPPC4 was overexpressed both starting from 24 h post-transfection (Fig. 5A).

### TRAPPC4 accumulation in the nucleus is associated with pERK1/2 staining in human colorectal cancers

**Figure 6:**
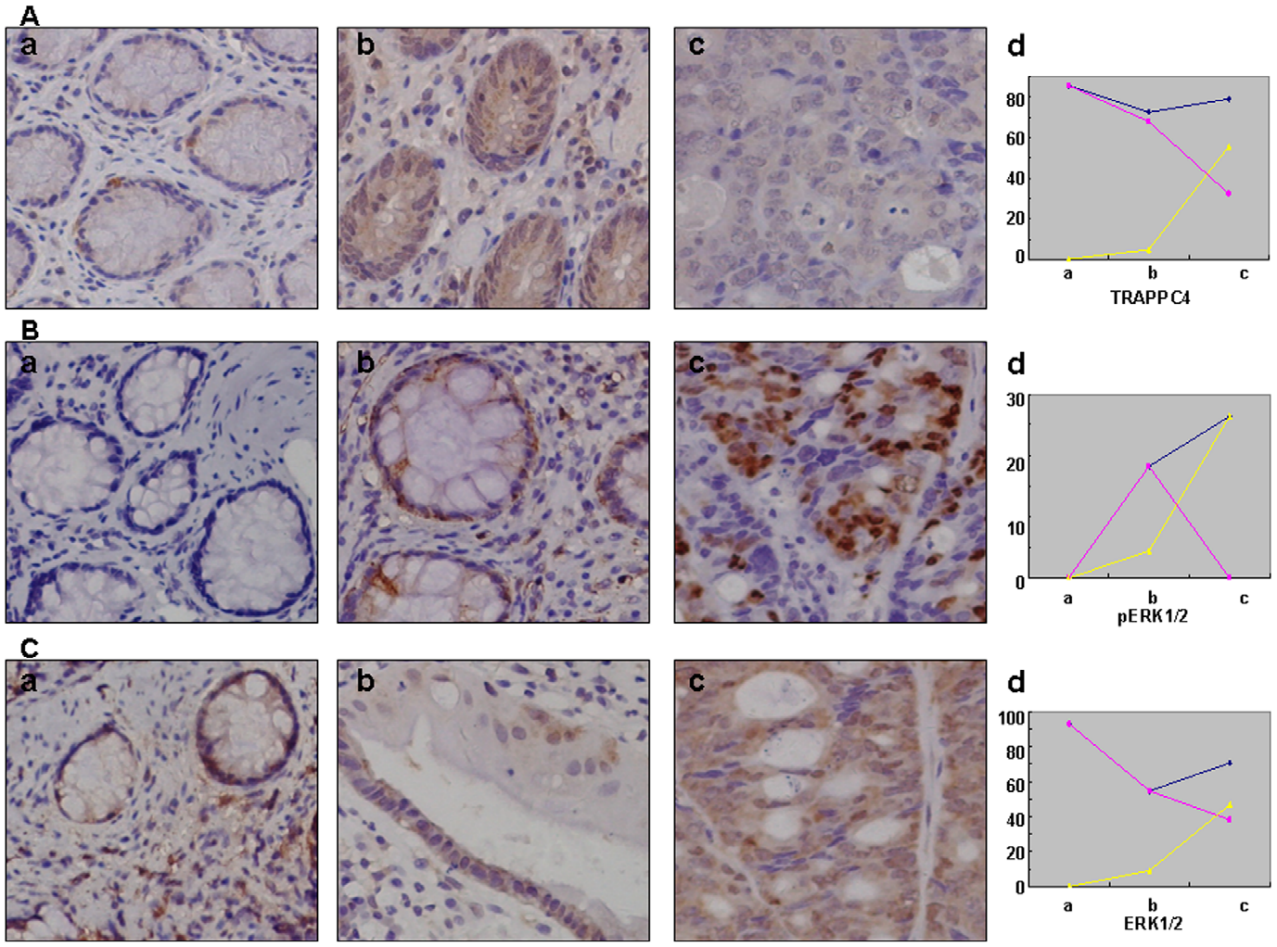
Representative immunohistochemical examination of TRAPPC4. (A), pERK1/2 (B) and ERK1/2 (C) in normal colonic epithelium (a), adenoma (b), and adenocarcinoma (c). Polygrams(d)show expression of the proteins in cytoplasm (pink), nucleus (yellow), and total (blue). A: Expression of TRAPPC4 has no significant difference in the three groups totally, but increases in nucleus from a to c. B: pERK1/2 mainly located in nucleus, and its level increases significantly from a to c. C: Expression of ERK1/2 has no significant difference in the three groups totally, but increases in nucleus from a to c. Original magnification ×200.

As little was known about the expression and localization of TRAPPC4 in colorectal cancer *in vivo*, we then compared its expression in normal colonic epithelium, adenoma and adenocarcinoma tissues by immunohistochemistry as described in Materials and methods. In normal colonic epithelium, TRAPPC4 staining was restricted to the cytoplasm (Fig. 6A). 4.55% of the cells in the adenoma samples showed nuclear staining, while it was 55.89% in the adencarcinoma samples (*p*<0.01). No significant differences were shown in total TRAPPC4 expression between the three groups (normal colonic epithelium group = 85.7%; adenoma group = 72.7%; adenocarcinoma group = 79.4%; *p*>0.05).(Tab. 1)

**Table 1:** Frequency of Expression of TRAPPC4, pERK1/2 and ERK1/2.

	Normal epithelium(%)	Adenoma(%)	Adenocarcinoma(%)
TRAPPC4			
Nucleus*	0 (0.0)	1 (4.5)	19 (55.9)
Cytoplasm*	12 (85.7)	15 (68.2)	11 (32.4)
Total*	12 (85.7)	16 (72.7)	27 (79.4)
pERK1/2			
Nucleus	0 (0.0)	1 (4.5)	9 (26.5)
Cytoplasm	0 (0.0)	4 (18.2)	0 (0.0)
Total	0 (0.0)	4 (18.2)	9 (26.5)
ERK1/2			
Nucleus	0(0.0)	2(9.1)	16(47.1)
Cytoplasm	13(92.9)	12(54.5)	13(38.2)
Total	13(92.9)	12(54.5)	24(70.6)
Number of samples	14	22	34

*Nucleus, nuclear staining; Cytoplasm, cytoplasmic staining; Total, staining in nucleus or cytoplasm or both.

Moreover, we analyzed the association between TRAPPC4 and pERK1/2 or ERK1/2 staining in normal colonic epithelium, adenoma and adenocarcinoma. While ERK1/2 staining was detected both in the cytoplasm and nucleus (Fig. 6C), its staining in the nucleus increased significantly in the adenocarcinoma group compared to the other two groups. Furthermore, the pERK1/2 activated proteins were all located in the nucleus, and there were significant differences among the three groups (Fig. 6B). Thus, our results demonstrate a significant correlation between nuclear TRAPPC4 expression and pERK1/2 levels (*r* = 0.996), and also between nuclear TRAPPC4 expression and ERK1/2 levels (*r* = 0.994).

## Discussion

The two components of the ERK1/2 complex are always found to be co-localized in colorectal carcinomas [17], [18], [19], [20], and the ERK1 and ERK2 MAPKs have attracted intense research interest because of their critical involvement in the regulation of cell proliferation and surviva [17]. Activated ERKs phosphorylate and regulate the activities of an ever growing roster of substrates that are estimated to comprise over 160 proteins [21]. Therefore, ERK1 and ERK2 were used as bait for two-hybrid-yeast screening in a human Hela cDNA library in the first step of this study. As a result, among the 23 binding proteins (11 with ERK2 and 12 with ERK1), the possible interaction of TRAPPC4 and ERK2 but not with ERK1 was revealed for the first time. Our co-immunoprecipitation and GST-pulldown experiments further confirmed TRAPPC4 as an ERK2 interaction partner.

Since little evidence of TRAPP proteins in CRC has been reported, and the role of the TRAPPC4-ERK2 interaction is still unknown, we first analyzed the effect of TRAPPC4 on phosphorylated ERK1/2, the activated form of ERK1/2. We found that, as a whole, overexpression of TRAPPC4 activated ERK1/2, while its depletion decreased pERK1/2 levels in the colon cancer-derived cell line SW1116. Thus, TRAPPC4 acts as a positive regulator for pERK1/2.

Although some are found in the cytoplasm, the majority of ERK substrates are nuclear proteins [22]. Activated ERKs can translocate to the nucleus where they phosphorylate and regulate various transcription factors, such as Ets family transcription factors, ultimately leading to changes in gene expression [23]. Further assessment of cytoplasmic and nuclear fractions showed that the location of pERK1/2 also altered along with TRAPPC4 expression levels. When TRAPPC4 was knocked down, the nuclear pERK1/2 level was notably down-regulated, but no significant difference was shown in the cytoplasm. When overexpressed, pERK1/2 expression increased both in the nucleus and cytoplasm, but more remarkably in the nucleus. Thus, TRAPPC4 is involved in not only the phosphorylation of ERK1/2 but also its spatial distribution in colorectal epithelial cells. TRAPP proteins are known to be part of a large multi-protein complex involved in ER-to-Golgi and intra-Golgi trafficking [5], [7], [8]. They present as several isoforms which differ in subunits, function, and location. For example, TRAPPCI is involved in the recruitment of ER-derived vesicles to the cis-Golgi [8], and TRAPPCII [7] is required for retrograde transport from the endosomes. Eva Loh *et al.* revealed that Bet3, the most conserved component of the TRAPP complex, is expressed in eight different mouse tissues, exists in two distinct pools in the cytosol and functions during ER-Golgi transport [10]. Therefore, TRAPPC4 may play a role in nucleocytoplasmic transport in colorectal cancer, but this mechanism will require additional study.

PDGF can signal through the ERK-MAPK pathway and activate the ERK cascade through receptor tyrosine kinases. We found that after treatment with PDGF, the effect of TRAPPC4 on pERK1/2 was completely or partially obscured. The results gave us an indication that TRAPPC4 may be involved in certain steps of PDFG-mediated activation of the ERK1/2 cascade. As for ERK1/2 phosphorylation, the effect of TRAPPC4 may not be as potent as that of PDGF. However, the detailed mechanism remains to be thoroughly investigated.

Moreover, we found that TRAPPC4 affected cell proliferation as well as cell death. Depletion of TRAPPC4 inhibited cell growth and induced apoptosis, while its overexpression contributed to cell growth. It was reported that activated ERK1/2 interacts with some substrates, such as Phosphoprotein enriched in astrocytes 15 (PEA-15),and Death associated protein kinase (DAPK), and is sequestered in the cytoplasm [24]. Deletion of PEA-15 markedly stimulates ERK-dependent proliferation and gene transcription; while PEA-15 overexpression blocks the proliferation via its ability to bind and sequester ERK1/2 in the cytoplasm [25]. DAPK sequesters ERK1/2 in the cytoplasm by interacting with ERK through a D-domain within its death domain. and the interaction promotes the proapoptotic function of DAPK [26]. Therefore, inhibition of ERK1/2 nuclear localization impairs ERK1/2-mediated survival signals and in addition augments the proapoptotic signals. In our study, after knockdown of TRAPPC4, the decrease in activation of ERK1/2 corresponded with less nuclear localization, which may ultimately lead to growth surpression and apoptosis. Whereas, more nuclear localization when TRAPPC4 overexpressed may promote cell growth.

The majority of the previous research on the TRAPP family was focused on yeast and normal mammalian cells or tissues [4], [5], [8], [10]. To our knowledge, only expression of TRAPPC2 has been investigated in the disease of SEDL [27]. To date, the location of TRAPP subunits has mainly been reported in the cytoplasm of mammalian cells [9], [10]. TRAPPC4 was reportedly located in spines of cultured hippocampal neurons [4], but little information on its role in disease has been obtained. Here, we analyzed TRAPPC4 expression in human normal colonic epithelium, adenoma, and adenocarcinoma tissues by immunohistochemistry. The results revealed that nuclear TRAPPC4 appears after normal epithelium tissue progresses to adenoma and adenocarcinoma. Thus, the alteration in localization of TRAPPC4 may be related to colorectal carcinogenesis. The question remains whether it may result from any gene mutations or structural modifications arising in TRAPPC4 during colorectal carcinogenesis. Meanwhile, pERK1/2, which was localized in the nucleus in our study, increased in adenocarcinoma compared to adenoma tissues and normal epithelial tissues. As mentioned above, the main function of the TRAPP family is in regulation of vesicular transport, and TRAPPC4 is involved in membrane trafficking in post-synaptic sites in neurons. One could ask if the mechanism of nuclear localization of TRAPPC4 in CRC is relevant to the transport of pERK1/2 from the cytoplasm to the nucleus. In fact, the TRAPP complex was shown to act as the guanine nucleotide exchange factor (GEF) for Ypt1 and Ypt31/32 GTPases both *in vitro* and *in vivo* [28], [29]. While Ypt1 GTPase is required at the cis-Golgi for the targeting and fusion of ER-derived vesicles [30], [31], the functions of the Ypt31/32 GTPases are also essential for the formation of trans-Golgi vesicles [32]. It remains to be seen whether a similar molecular mechanism exists for TRAPPC4 in CRC in yet further studies.

**Figure 7:**
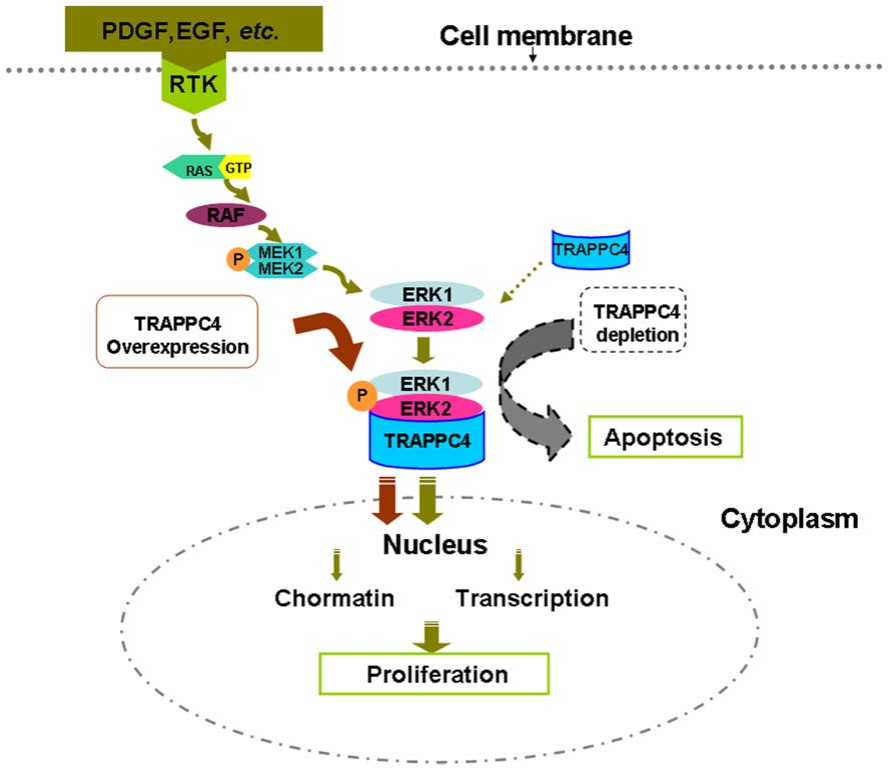
The speculated role of TRAPPC4-ERK2 interaction in CRC. Extracellular signals, such as PDGF, activate ERK cascades(RAS/RAF/MER/ERK) through receptor tyrosine kinases (RTKs) and utimately regulate cell proliferation and apoptosis. TRAPPC4 binds and activates ERK1/2 as well as participates in the nuclear transport of pERK1/2. When TRAPPC4 is depleted, less ERK1/2 is phosphorylated, and relatively more pERK1/2 remains in the cytoplasm, which leads to suppression of cell proliferation and apoptosis. When TRAPPC4 is overexpressed, more ERK1/2 is activated, and even more is transported into the nucleus, finally leading to increased cell growth.

In conclusion, our study demonstrated the interaction of TRAPPC4 with ERK2. In colorectal cancer, it not only regulates the activation of ERK1/2 but also affects the distribution of activated ERK1/2. It modulates cell proliferation and apoptosis in CRC cells. In consideration with previous studies, we speculate that, in CRC, TRAPPC4 binds and activates ERK1/2 as well as participates in the nuclear transport of pERK1/2. When TRAPPC4 is depleted, less ERK1/2 is phosphorylated, and relatively more pERK1/2 remains in the cytoplasm, which leads to suppression of cell proliferation and apoptosis. When TRAPPC4 is overexpressed, more ERK1/2 is activated, and even more is transported into the nucleus, finally leading to increased cell growth (Fig. 7). Future studies will be required to clarify this proposed molecular mechanism.

## Materials and Methods

### Plasmids and transfections

The full-length ERK1, ERK2, and TRAPPC4 cDNA were obtained by PCR from a human cDNA library. For yeast two-hybrid assays, full-length fragments of ERK1 and ERK2 cDNAs were cloned both in frame with the *Sfi*I site in the pGB plasmid. For binding assays, the full-length TRAPPC4 cDNA was cloned in pCDEF-Myc, and full-length ERK2 cDNA was cloned in pCDEF-Flag. ERK2 was subcloned in pGEX-5x to produce glutathione *S*-transferase (GST) fusion proteins, and TRAPPC4 was subcloned in pET-28a to produce His fusion proteins.

For the co-immunoprecipitation experiments below, 1.6×10^6^ cells/well of 6-cm dishes were co-transfected with vectors pCDEF-Myc-TRAPPC4 and pCDEF-Flag-ERK2 expressing TRAPPC and ERK2, respectively, using a plasmid: transfection reagent ratio of 1∶4 (4 µg each plasmid). Empty vector controls were pCDEF-Myc or pCDEF-Flag (both from Shanghai Genomics).

### Yeast two-hybrid assays

Yeast two-hybrid screening was performed to identify ERK1 and ERK2 interacting proteins by using the Clontech Matchmaker Two-Hybrid System (Cat. #K1612-1) according to the manufacturer's instructions. Bait plasmids PGB-ERK1-G and PGB-ERK2-G were transformed into the yeast strain Y190. Toxicity effects and self-activation tests were performed using a β-galactosidase assay. Yeast transformed with bait plasmids were cultured and screened with the human HeLa Matchmaker cDNA library (Clontech, CAT#HL4048AH) and then grown on Leu-Trp-His plates for selection and validation using the ß-galactosidase assay. Plasmids from positive colonies were extracted, amplified in bacteria and co-transformed back into yeast along with the bait plasmids for further testing in ß-galactosidase assays. Plasmids of positive hits were sequenced and analyzed.

### Co-immunoprecipitation (co-IP) and GST-pulldown analysis

Co-immunoprecipitation was performed as described previously [33]. Both the input and IP samples were analyzed by Western blot using various antibodies at the following dilutions: Flag Antibody (1∶1000), Myc Antibody (1∶1000) Flag-HRP Antibody (1∶4000), Myc-HRP Antibody (1∶2000) (all from Shanghai Genomics, Shanghai, China), and goat anti-mouse IgG-HRP Antibody (1∶5000) (Santa Cruz Biotechnology, Santa Cruz, U.S.A.). Co-transfection of expression vectors for p16 and TSSK1 (Shanghai Genomics, Shanghai, China) was used for the positive control co-immunoprecipitation.

GST protein and GST-ERK2 fusion proteins were expressed and purified according to manufacturer's instructions (GE Healthcare, London, U.K.). For the pull-down assay, 1–5 mg of the GST or GST fusion proteins were mixed with 40 ml of a 50% suspension of glutathione-Sepharose 4B beads for 2 h in binding buffer [25 mM HEPES-NaOH (pH 7.5), 12.5 mM MgCl_2_ 10% Glycerol, 5 mM DTT, 0.1% NP-40, 150 mM KCl and 20 mM ZnCl2]. Then 1–5 mg of 6×His-TRAPPC4 fusion proteins was added followed by incubation for another 2 h. The pellets were washed extensively, and were identified by western blotting with 6×His Antibody (1∶1000) (Abcam, Cambridge, U.K.).

### Cell culture

The colon cancer-derived cell line SW1116 (purchased from the Cell Bank of Type Culture Collection of Chinese Academy of Sciences, Shanghai Institute of Cell Biology, Chinese Academy of Sciences) was maintained by serial passages in RPMI 1640 containing 10% heat-inactivated fetal calf serum (FCS), 100 units/ml penicillin and 100 µg/ml streptomycin. Human 293T cells (purchased from the Cell Bank of Type Culture Collection of Chinese Academy of Sciences, Shanghai Institute of Cell Biology, Chinese Academy of Sciences) were maintained in DMEM supplemented with 10% FCS and antibiotics. Cultures were incubated at 37°C in standard tissue culture incubators as previously described (5). A total of 106 cells were plated per 100-mm dish. Platelet-derived growth factor (PDGF) (Sigm-Aldrich, St. Louis, U.S.A.) was used to sitimulated SW1116 cells at a final concentration of 10 ng/ml for 15 min.

### RNAi and transient transfections

TRAPPC4 expression was inhibited using ON-TARGETplus SMARTpool siRNA and Dharma*FECT* transfection reagent (Thermo Fisher Science, Inc., Rockfrd, USA) according to the manufacturer's instructions. Briefly, SW1116 were seeded in 12-well plates in medium containing 10% serum at a density that would allow cells to reach 50% confluence on the day of transfection. The transient transfections were performed using 100 nM of each siRNA duplex. siGLO RISC-Free Control siRNA was used as a negative control. The selective silencing of TRAPPC4 was confirmed by Western blot analysis.

### Western blot assays

Western blot assays were performed to examine the phosphorylation of ERK1/2 and expressions of total ERK1/2 and TRAPPC4 proteins. Cytoplasmic and nuclear extracts were prepared with NE-PER® Nuclear and Cytoplasmic Extraction Reagents (Thermo Fisher Scientific, Inc., Rockfrd, USA )according to the manufacturer's instructions, and whole cell extracts were prepared by a previously described method [34] from both siRNA-treated TRAPPC4-plasmid treated and mock-treated, or transfected and mock-transfected SW1116 cells. After electrophoresis, 400 µg of proteins were electroeluted at 120 volts onto a polyvinylidene difluoride membrane (Invitrogen, California, U.S.A.). Primary antibodies raised against phosphorylated ERK1/2 (Thr202/204) and ERK1/2 were purchased from Cell Signaling Technology (Danvers, U.S.A.). The anti-TRAPPC4 antibody was purchased from Sigm-Aldrich (St. Louis, U.S.A.), and the GAPDH (Sigm-Aldrich, St. Louis, U.S.A.) antibody was used as a control for total and cytoplasmic protein input. An antibody against TATA box binding protein (TBP) (Sigm-Aldrich, St. Louis, U.S.A.) was used as a control for nuclear protein input. The Western blotting analysis was repeated at least three times.

### Cell viability assays

The SW1116 cells were seeded onto 96-well plates at 2000 cells/well, cultured for 24 h, and transfected with TRAPPC4 and control siRNA. Cell proliferation was measured using the Cell Counting Kit-8 (CCK-8) [35]. At each time point, the SW1116 cells were incubated with 10 µl CCK-8 (Dojindo Laboratories, Kumamoto, Japan) per well (100 µl medium/well) for 1 h at 37°C, 5% CO_2_. The absorbance was measured at 450 nm. Data were expressed as the percentage of viable cells as follows: relative viability (%) = [A450(treated)−A450(blank)]/[A450(control)−A450(blank)]×100%.

### Apoptosis assays

Apoptosis was carried out using the Annexin V assay with Propidium iodide (PI) counterstaining (Biosea Biotechnology Co., LTD, Beijing, China) allowing quantification by flow cytometry. In brief, after treated with TRAPPC4 siRNA and Control siRNA for 48 h, both floating and trypsinized adherent cells (1×10^6^) were collected and resuspended in 500 µl of binding buffer containing 10 µl of annexin-V fluorescein isothiocyanate and 5 µl of PI, then incubated for 15 minutes in the dark at room temperature. Analysis was immediately performed using a flow cytometer.

### Tissue microarray and immunohistochemical staining for CRC and colorectal adenoma tissues

All specimens were from patients (34 primary colorectal adenocarcinomas and 22 adenomas) who underwent surgery at the Shanghai Renji Hospital from July 2006 to January 2008. The protocol had the approval of the Ethics Committee of the Shanghai Jiao-Tong University School of Medicine, Renji Hospital, and the research was carried out according to the provisions of the Helsinki Declaration of 1975. Written informed consent was obtained from all participants involved in the study. Meanwhile, 14 specimens of normal colonic epithelium taken from patients without colorectal cancer were used as the controls. A tissue microarray (diameter, 1.0 mm; depth, 4 µm) was prepared by Outdo Biotech (Shanghai, China) using standard techniques. The tissue microarray sections were deparaffinized in xylene and rehydrated using a graded series of ethanol. A three-step streptavidin-biotin horseradish peroxidase method was used, and the expressions of ERK1/2, pERK1/2, and TRAPPC4 were examined with the primary antibodies [ERK1/2, dilution 1∶100; pERK1/2 (Thr202/Tyr204), dilution 1∶100, TRAPPC4, dilution 1∶50] using the LSAB+ kit (DakoCytomation, Copenhagen, Denmark) according to the manufacturer's instructions. The slides were examined independently by two investigators blinded to both clinical and pathologic data. Protein expression was quantified based on both staining intensity, and positive frequency according to a previously described scoring method [36].

### Statistical analysis

The data from least 3 independent experiments performed in triplicate are presented as means ± standard deviation (SD). Comparisons between groups were performed using the Student's paired *t* test or chi-square test (for immunohistochemistry part only). Differences in means with a value of *p*<0.05 was considered significant.
